# The ability of dietary essential oils to mitigate nickel-induced growth retardation, immune-antioxidant suppression, and endoplasmic reticulum stress activation in Nile tilapia

**DOI:** 10.1007/s10695-025-01482-2

**Published:** 2025-03-31

**Authors:** Shaimaa A. A. Ahmed, Ghada I. Abd El-Rahman, Haiam A. Mohammed, Samar A. Abdo, Mohamed Y. M. Aly, Hala Elshahat Ghannam, Fatma Mahsoub, Tarek Khamis, Rowida E. Ibrahim

**Affiliations:** 1 Department of Aquatic Animal Medicine, Faculty of Veterinary Medicine, Zagazig University, Zagazig, 44511 Egypt; 2 Department of Clinical Pathology, Faculty of Veterinary Medicine, Zagazig University, P.O. Box 44511, Zagazig, Egypt; 3 Physiology Department, Faculty of Veterinary Medicine, Zagazig University, P.O. Box 44511, Zagazig, Egypt; 4 Biochemistry Department, Faculty of Veterinary Medicine, Zagazig University, P.O. Box 44511, Zagazig, Egypt; 5 Pollution Laboratory, Freshwater and Lakes Division, National Institute of Oceanography and Fisheries (NIOF), Cairo, Egypt; 6 Department of Animal and Poultry Production, Faculty of Technology and Development, Zagazig University, Zagazig, Egypt; 7 Department of Pharmacology, Faculty of Veterinary Medicine, Zagazig University, Zagazig, 44511 Egypt

**Keywords:** Endoplasmic reticulum stress, Histopathology, Nickel, *Oreochromis niloticus*, Tea tree and basil oils

## Abstract

Fish immunity is significantly impacted by waterborne metal intoxication. Nickel is a ubiquitous metal in aquatic bodies which badly impacts fish immune responses and survival. In the current research, we looked into the possible protective effects of essential oils of tea tree (TTO) and basil (BEO) against nickel exposure in Nile tilapia, or *Oreochromis niloticus*. To achieve this, 240 Nile tilapia (27.92 ± 0.22 g) were categorized into six groups for 45 days; three groups were fed on basal control, TTO, and BEO diets without Ni exposure. The other three groups were subjected to Ni at a level of 3.6 mg/L and fed on basal, TTO, and BEO diets. The outcomes showed a substantial decrease in the growth measures and survival % in the Ni-exposed fish. Oxidative stress (higher splenic malondialdehyde and lower splenic catalase, reduced glutathione, and superoxide dismutase), immune suppression (lower serum lysozyme, myeloperoxidase, and nitric oxide), and elevated serum cortisol levels were observed in the Ni-intoxicated group. The Ni-intoxicated group exhibited various pathological alterations detected in the intestinal tissue mainly inflammatory and necrotic changes, moreover, moderate depletion of lymphoid elements represented by necrotic lymphocytes in the splenic tissue. Ni-exposure induced endoplasmic reticulum stress through up-regulation of the splenic expression of protein kinase R-like endoplasmic reticulum kinase, activating transcription factor 6, CCAAT/enhancer-binding protein homologous protein, X-box binding protein 1, α-subunit of eukaryotic initiation factor 2, inositol-requiring kinase 1a, mitogen-activated protein kinase, c-JunN-terminal kinase, and binding protein for immunoglobulins. Feeding on the TTO and BEO diets improved the growth measures, survival, and antioxidant capacity. Immunomodulation, enhancement of the intestinal and splenic architecture, and relief of the endoplasmic reticulum stress condition were noticed when the Ni-intoxicant groups were fed on TTO and BEO diets. Overall, the effect of the TTO diet was more pronounced than the BEO diet in mitigating the negative consequences of Ni-intoxication in Nile tilapia. Dietary fortification of 0.1% TTO and/or 0.1% BEO is recommended during Ni exposure in aquaculture practices.

**Supplementary Information:**

The online version contains supplementary material available at 10.1007/s10695-025-01482-2.

## Introduction

Fish immune responses are altered as a result of a variety of environmental challenges. Heavy metals can alter specific biochemical variables and innate and adaptive immune functions, which have an immediate or long-term impact on fish health (Borgia et al. 2018). Heavy metals can cause negative impacts on the fish body by generating oxygen reactive species (ROS), which lead to oxidative stress damage of DNA, and change the expression patterns of crucial proteins, hormones, and enzymes (Shahjahan et al. 2022).

Nickel (Ni) is a ubiquitous, naturally occurring trace metal found in high concentrations in waterways (Topal et al. 2017). According to Topal et al. (2015), the most common form of nickel that escapes from both natural sources and human activity is nickel chloride (NiCl_2_). Ni-exposed fish have been reported to exhibit a variety of disorders, including decreased locomotor activity, skin abnormalities, delayed hatching, deformities in the development of the muscles and gills, and elevated mortality (Wang et al. 2020; Defo et al. 2014). Ni exposure retarded growth and induced pathological changes in the architecture and functions of the splenic, hepatic, and renal tissue in Nile tilapia (*Oreochromis niloticus*). In addition, inhibited the antioxidant-immune functions of the fish (El-Houseiny et al. 2022). Dietary approaches that can both improve the well-being and growth of cultured fish and lessen the harmful effects of waterborne contaminants are desperately needed in the aquaculture sector these days (Edrees et al. 2024). Regarding this, herbal medicine interest in using them as aquafeed additives has increased, especially with heightened concern about chemical additives and antibiotics around the world (Abdel Rahman et al. 2023).

Tea tree essential oil (TTO) is produced from the *Melaleuca alternifolia* leaves and branches (Pazyar et al. 2013) and has a majority of alcohols and monoterpenes (Hammer et al. 2012). TTO has anti-microbial properties that include the ability to damage the cell membranes of bacteria and fungi, prevent the growth of fungal spores, and obstruct the reproduction of viruses in cells (Garozzo et al. 2011; Li et al. 2016, 2017). A prior study in aquaculture demonstrated that increasing the diet’s 250 mg/kg TTO content could increase *O. niloticus*’s intestinal villus height (Valladão et al. 2017). The growth and antioxidant-immune capacity of freshwater prawn (*Macrobrachium rosenbergii*) were enhanced by dietary supplementation of 100 mg/kg TTO (Liu et al. 2022a, b). In *Aeromonas hydrophila*-infected silver catfish (*Rhamdia quelen*), TTO reduced histopathological damage to the gills and enhanced the immune system (Baldissera et al. 2017; Souza et al. 2016).

Basil essential oil (BEO) is extracted from the fresh leaves of *Ocimum basilicum* (*Lamiaceae* family) (Sajjadi 2006). Because BEO is readily available, affordable, and environmentally friendly, it can be widely used as a food flavoring agent and in the medical, cosmetic, and perfumery fields (Telci et al. 2006). BEO has several active ingredients, including flavonoids, triterpenoids, phenolics, essential oils, glycosides, eugenol, methyl chavicol, methyl cinnamate, and bergamotene (Bihari et al. 2011; Bilal et al. 2012). BEO displayed immunomodulatory and antioxidant properties as well as a protective effect against water-born copper intoxication in *O. niloticus* as a result of its active ingredients (Ahmed et al. 2022). Consequently, we designed this work to explore the protective potential of TTO and/or BEO against water-born Ni intoxication in Nile tilapia. For this purpose, the antioxidant-immune indices, splenic immune cascades, and histological assays were investigated.

## Materials and methods

### Tested compounds

NiCl_2_ (NiCl_2_0.6 H_2_O, extra pure 99%, molecular weight = 237.69) was purchased from Alpha Chemica Company (Mumbai, India). Other chemicals were purchased from Sigma-Aldrich, St. Louis, MO, USA, and were highly analytical.

### Fish and housing condition

Nile tilapia (27.92 ± 0.22 g) was supplied from the Fish Research Unit (Faculty of Veterinary Medicine, Zagazig University, Egypt). The fish was subjected to clinical examination for health status assurance following CCAC (2005) guidelines. The fish were housed in glass aquaria (100 L capacity) and received a basal diet for 15 days before the study. The aquaria’s water quality parameters were measured following APHA (2005), such as pH (6.8 ± 0.6), temperature (27.9 ± 0.55 ◦C), ammonia (0.024 ± 0.01 mg/L), and dissolved oxygen (6.50 ± 0.2 mg/L), were all under similar conditions.

### Diets and study setup

Three experimental diets were formulated to meet the nutritional needs of Nile tilapia following the NRC (2011) recommendations. The first diet was control basal (Table 1) while the second and third diets were basal diets with the addition of 0.1% TTO and 0.1% BEO. The dietary levels for TTO and BEO were selected based on the recommendations of Liu et al. (2022c) and de Souza et al. (2019), respectively. Each diet component was mixed thoroughly and then pelleted using the pelleting machine (Fish Research Unit, Zagazig University, Egypt). After that, the pellets were maintained to air-dry at room temperature (25 °C) with continuous rotation to ensure efficient dryness. Ultimately, the pellets were placed in tight plastic bags and refrigerated until they were needed.

**Table 1:** Physical and chemical composition of the basal diet (g/kg)

Feed ingredients	(g/kg)
Fish meal	103.90
Soybean meal	300
Corn gluten meal	100
Yellow corn	351.60
Wheat bran	100
Sunflower oil	20.5
Limestone	11
Common salt	1.5
Mineral and vitamin premix^a^	5
Choline chloride	1
Vitamin C	0.5
Carboxymethyl cellulose	5
**Chemical composition (analyzed)**	
Digestible energy, kcal/kg	3063.46
Crude protein	301.72
Crude fiber	45.4
Ether extract	58
Nitrogen free extract	439.58
Methionine	6.4
Lysine	18.1
Calcium	6.4
Phosphorus	5
Sodium	1.3

^a^Mineral and vitamin premix (NEOFARMA). Each kg contains vitamin A acetate (6,250,000, IU), vitamin D3 (cholecalciferol, 2,500,000 IU), vitamin E (α-tocoopherol, 25,000 mg), vitamin K3 (menadione sodium bisulfate, 1750 mg), vitamin B1 (500 mg), vitamin B2 (2750 mg), vitamin B6 (1250 mg), vitamin B12 (10 mg), nicotinic acid (niacin, 20,000 mg), calcium pantothenate (5000 mg), folic acid (500 mg), biotin (50 mg), iron sulfate (22,000 mg), manganese oxide (31,000 mg), copper sulfate (2500 mg), zinc oxide (37,500 mg), potassium iodide (650 mg), selenium selenite (113 mg), cobaltous sulfate (50 mg), ethoxyquin (250 mg), wheat bran (carrier, 120 gm), and limestone (carrier, up to 1 kg)

For 45 days, 240 Nile tilapia were divided into six groups, each containing four replicates (40 fish per group, 10 fish per replicate). The C (control), TTO, and BEO groups were fed on a basal diet, basal diet with TTO, and basal diet with BEO, respectively without Ni exposure. While, the Ni, Ni + TTO, and Ni + BEO groups were exposed to 1/10 of 96-h lethal concentration 50 of Ni which equals 3.6 mg/L based on the protocol of El-Houseiny et al. (2022). The experimental groups were fed three times daily till satiation. The waste materials were daily disposed of from the aquarium with complete water evacuation and changed twice weekly, during which fresh Ni solution was added to maintain the Ni level constant during the experimental period. During the trial time, the clinical symptoms and mortality were registered daily in all fish groups.

### Growth measurements

Benzocaine solution (100 mg/L) was applied for fish sedation and reduction of stress during the weighting procedure (Neiffer and Stamper 2009). The fish’s initial weight (IW) and final weight (FW) were calculated at the start and end of the trial. During the trial, the feed intake (FI) was determined through subtraction of the refused pellets from the offered ones. The other growth measurements (weight gain (WG %), feed conversion ratio (FCR), and specific growth rate (SGR %)) were calculated as follows:

$$\begin{array}{l}\mathrm{WG}\;(\%)\:=\:100\:\times\:(\mathrm{FW}-\mathrm{IW})/\mathrm{IW}\\\mathrm{FCR}\:=\:\mathrm{FI}\;(\mathrm g)/\mathrm{WG}\;(\mathrm g)\\\mathrm{SGR}\;\%\:=\:\lbrack(\mathrm{In}\;\mathrm{FW}-\;\mathrm{In}\;\mathrm{IW})/\mathrm{time}\;(\mathrm{days})\rbrack\:\times\:100\\\mathrm{Survival}\;(\%)\:=\:(\mathrm{number}\;\mathrm{of}\;\mathrm{fish}\;\mathrm{in}\;\mathrm{each}\;\mathrm{group}\;\mathrm{remaining}\;\mathrm{after}\;\mathrm{the}\;45-\mathrm{day}\;\mathrm{feeding}\;\mathrm{period}/\mathrm{initial}\;\mathrm{number}\;\mathrm{of}\;\mathrm{fish})\:\times\:100\end{array}$$

### Blood and tissue sampling

Fish were fasted for a full day, and then blood was drawn from eight fish in each group after 45 days of exposure. Initially, a benzocaine solution (100 mg/L) was applied to anesthetize the fish (Neiffer and Stamper 2009). From the caudal blood vessels, blood samples were obtained to obtain the serum after 10 min of centrifuging the sample at 1500 *g*. After that, the serum was maintained at − 20 °C to assess the immunological indices. Afterward, the same fish that was used for blood collection were killed by an overdose of benzocaine solution (400 mg/L) (Tran-Duy et al. 2008) and spleen tissues (8/group) were taken for oxidant/antioxidant indices and transcriptomic analysis. The spleen samples for gene expression were preserved in 1 mL of QIAzol (Qiagen, Germany) and stored at − 80 °C until an assay was performed. Additionally, intestine and spleen tissues (8/group) were sampled and kept in 10% neutral buffered formalin for histological analysis.

### Oxidant-antioxidant indices

The lipid peroxides biomarker (Malondialdehyde, MDA) and antioxidant indices [catalase (CAT), reduced glutathione (GSH), and superoxide dismutase (SOD)) were determined in the spleen tissue. The spleen tissues were homogenized using buffer (pH 7.4); after that, centrifugation of the homogenates at 10,000 g for 20 min at 4 °C was carried out. After that, the supernatant was collected for evaluation of oxidant-antioxidant indices. MDA (Catalog No. MD2529), CAT (Catalog No. CA2517), GSH (Catalog No. TA2511), and SOD (Catalog No. SD2521) were measured spectrophotometry using the Bio-Diagnostic kits (Cairo, Egypt).

### Immune indices and stress marker

The manufacturer’s recommendations were followed for the assessment of serum lysozyme (LYZ) (Catalog. No. EK750008AFG; Bioscience, Northbrook, IL, USA) and serum myeloperoxidase (MPO) level (Catalog. No. MBS016324; MyBioSource, USA) and were measured. The serum nitric oxide (NO) was assessed spectrophotometrically according to the protocol of Bryan and Grisham (2007). The Saliu et al. (2017) protocol was followed to estimate the serum cortisol level utilizing the kit (BioQuoChem) at an optical density of 450 nm.

### Histopathological study

Specimens from the intestine and spleen were marinated in 10% neutral buffered formalin, subsequently dehydrated in upward grades of alcohol, cleared in xylene, and embedded in paraffin to create blocks that were sliced into 5 µm sections. Then, the paraffin sections were collected and stained with hematoxylin and eosin (H&E) according to Suvarna et al. (2018), examined, and photographed under a light microscope (AmScope, Irvine, CA, USA).

### Gene expression analysis

QIAzol (Qiagen, Germany) was utilized to get total RNA from splenic samples (50 mg) based on the manufacturer’s directions. The agarose gel electrophoresis and spectrophotometer (BioRad, CA, USA) were used to measure the concentration and purity of the extracted RNA. The recovered RNA was treated with DNase (Takara, Shiga, Japan) to remove any possible DNA contaminants. To obtain the complementary DNA (cDNA), a reverse transcriptase kit (Applied Biosystem, California, USA) was used relying on the manufacturer’s directions. The primers from Sangon Biotech, Beijing, China, were utilized to analyze the following genes using real-time quantitative PCR (RT-qPCR): protein kinase R-like endoplasmic reticulum kinase (*PERK*), activating transcription factor 6 (*ATF-6*), CCAAT/enhancer-binding protein homologous protein (*CHOP*), X-box binding protein 1 (*XBP-1*), α-subunit of eukaryotic initiation factor 2 (*EIF-2a*), inositol-requiring kinase 1a (*IRE-1a*), mitogen-activated protein kinase (*MAPK-1*), c-JunN-terminal kinase (*JNK*), and binding protein for immunoglobulins (*BIP*) (Table 2).

**Table 2:** Primers for the real-time quantitative PCR amplification

Gene	Sequence	Tm	Primer efficiency	Size	Accession no
*PERK*	GATCGAGATGCCGATGCAGA	60.04	94.5	91	XM_003447769.5
	TGTGTGACGAGGACGAACTG	59.97			
*ATF-6*	TGGTATGAGAGGTCGCTGGA	60.03	93.45	150	XM_003440029.5
*CHOP*	GACACAGGAGGGGCAAAACT	60.18			
	GCTCCTTCTGGAAGCACAAA	58.39	98.5	81	XM_013275519.3
	TCGATGGAGATGATGCCTGC	59.97			
*XBP-1*	GGTGGAGGAGGAGACCATCT	60.03	95.45	198	XM_013276886.2
	ATGTCGCTGAAAGGGGAAGG	60.04			
*EIF-2a*	TTCGACTCCATCGCCAACAA	59.97	96.54	139	XM_005462748.4
	GGTACTGATACAGGCGGACG	59.97			
*IRE-1a*	AAGCAGGAAGGAAACGCTGA	59.89	97.5	197	XM_025905478.1
	CTTGCCCTCCCAGTCACATT	59.96			
*MAPK-1*	CTTTGGTTTGGCTCGTGTGG	59.97	98.45	171	XM_003444474.5
	TTGGACAGCATCTCAGCCAG	60.04			
*JNK*	AAAGCGTGGTGGAGTCTCTG	60.03	97.82	104	XM_005455390.4
	CTCCCTCTCAGCCTCTTCCT	59.97			
*BIP*	GTCGAGCAGATTGGAGAGCA	59.83	99.64	116	XM_019361056.2
	CAGTCCCCACGTTCTCCTTC	60.04			
*Gapdh*	GATAATGGCAAACTTGTCGTCG	58.33	99.53	205	NM_001279552.1
	ACATTGGAGCATCGGGTGAG	60.11			

*PERK*, protein kinase R-like endoplasmic reticulum kinase; *ATF-6*, activating transcription factor 6; *CHOP*, CCAAT/enhancer-binding protein homologous protein; *XBP-1*, X-box binding protein 1; *EIF-2a*, α-subunit of eukaryotic initiation factor 2; *IRE-1a*, inositol-requiring kinase 1a; *MAPK-1*, mitogen-activated protein kinase; *JNK*, c-JunN-terminal kinase; *BIP*, binding protein for immunoglobulins; *Gapdh*, glyceraldehyde3-phosphate dehydrogenase;

*Tm*, melting temperature

The RT-qPCR conditions were kept for 40 cycles of 95 °C for 10 s and 60 °C for 15 s, with an in-between dissociation analysis step with initial denaturation temperature (95 °C for 10 min). The gene expression data was evaluated following the 2^−ΔΔCT^ protocol (Schmittgen and Livak 2008) using glyceraldehyde3-phosphate dehydrogenase (*Gapdh*) as a housekeeping gene.

### Statistical analysis

The Shapiro–Wilk test was applied to test the data normal distribution. A one-way analysis of variance (ANOVA) was applied to search for any noteworthy variations between groups. Tukey multiple range tests were used to analyze the difference between means. For the statistical analysis, SPSS Version 17 for Windows (SPSS Inc., Chicago, IL, USA) was utilized. The data was described using the means + standard error (SE).

## Results

### Clinical signs and survival

The C, TTO, and BEO groups exhibited 100% survivability at the end of the exposure time, while the Ni group recorded the lowest significant survivability (*P* = 0.001). The survival of the TTO (90%) and BEO (87.50%) groups was significantly enhanced compared to the Ni group (77.50%) (Table 3). The Ni-exposed fish suffered from skin darkness and fin rot (Supplementary Fig. 1), as well as lethargic swimming.

**Table 3:** Growth indices and survival % of Nile tilapia exposed to water-born nickel (Ni) and fed tea tree and basil essential oils for 45 days

	IW (g/fish)	FW (g/fish)	WG (%)	FI (g/fish)	FCR	SGR (%/day)	Survival (%)
C	27.89 ± 0.10	52.63 ± 0.40^c^	88.70 ± 0.33^c^	63.66 ± 0.53^a^	2.57 ± 0.01^c^	0.61 ± 0.01^c^	100 ± 0.00^a^
Ni	27.86 ± 0.17	38.09 ± 0.37^f^	36.72 ± 0.20^f^	36.00 ± 0.57^c^	3.52 ± 0.14^a^	0.31 ± 0.01^f^	77.50 ± 4.78^d^
TTO	27.85 ± 0.13	58.32 ± 0.29^a^	109.40 ± 0.28^a^	62.66 ± 0.64^a^	2.06 ± 0.02^e^	0.72 ± 0.02^a^	100 ± 0.00^a^
BEO	27.91 ± 0.23	57.06 ± 0.44^b^	104.44 ± 0.29^b^	63.33 ± 0.73^a^	2.17 ± 0.01^d^	0.69 ± 0.03^b^	100 ± 0.00^a^
Ni + TTO	27.96 ± 0.08	45.24 ± 0.53^d^	61.80 ± 0.46^d^	52.33 ± 0.66^b^	3.03 ± 0.10^b^	0.46 ± 0.01^d^	90 ± 5.77^b^
Ni + BEO	28.05 ± 0.09	42.66 ± 0.32^e^	52.08 ± 0.35^e^	53.33 ± 0.33^b^	3.65 ± 0.09^a^	0.40 ± 0.02^e^	87.50 ± 7.50^c^
*P* value	0.93	< 0.001	< 0.001	< 0.001	< 0.001	< 0.001	0.001

Values not sharing the same superscript letter in the same column are significantly different (*P* < 0.05; one-way ANOVA; *n* = 4). *IW*, initial weight; *FW*, final weight; *WG %*, weight gain percent; *FI*, feed intake; *FCR*, feed conversion ratio; *SGR*, specific growth rate. C (control), TTO, and BES: fish groups fed on basal, tea tree, and basil essential oils diets, respectively, without toxicant exposure. Ni, Ni + TTO, and Ni + BEO: fish groups exposed to 3.6 mg/L nickel and fed on basal, tea tree, and basil essential oils diets, respectively

### Growth measures

The growth measures including FW, WG %, and SGR were considerably increased (*P* < 0.001) in the TTO group accompanied by the BEO group comparable to the C group (Table 3). These measures were considerably lowered in the Ni group in contrast to the C group and were enhanced when the Ni group fed on the TTO diet (Ni + TTO) followed by the BEO diet (Ni + BEO) in comparison to the C group. No considerable difference in the FI value among the C, TTO, and BEO groups, while this value was significantly lowered (*P* < 0.001) in the Ni group in comparison to the C group and improved in the Ni + TTO and Ni + BEO groups when contrasted to the Ni group. The FCR value was lower (*P* < 0.001) in the TTO accompanied by the BEO group comparable to the C group, while this value was higher in the Ni and Ni + BEO groups followed by the Ni + TTO group comparable to the C group.

### Oxidant/antioxidant indices

The MDA level was substantially lowered (*P* < 0.001) with increased CAT, GSH, and SOD (*P* < 0.001) activity in the TTO and BEO groups contrasted to the C group (Table 4). The Ni group had a considerably high MDA level with the lowest antioxidant enzyme activity in contrast to the C group. The MDA level was substantially modulated with a substantial enhancement of the antioxidant enzyme activity in the Ni + TTO group accompanied by the Ni + BEO group in contrast to the Ni group.

**Table 4:** Oxidant-antioxidant indices of Nile tilapia exposed to water-born nickel (Ni) and fed tea tree and basil essential oils for 45 days

	MDA (µmol/g)	CAT (U/mg)	GSH (U/mg)	SOD (U/mg)
C	5.26 ± 0.08^d^	17.54 ± 0.39^c^	84.42 ± 0.78^c^	36.60 ± 0.45^a^
Ni	10.36 ± 0.22^a^	4.56 ± 0.22^f^	47.44 ± 0.73^f^	13.00 ± 0.52^c^
TTO	2.99 ± 0.07^e^	22.21 ± 0.59^a^	115.38 ± 0.71^a^	38.64 ± 0.41^a^
BEO	3.51 ± 0.15^e^	20.10 ± 0.38^b^	102.61 ± 0.47^b^	38.08 ± 0.28^a^
Ni + TTO	6.60 ± 0.28^c^	12.70 ± 0.21^d^	78.52 ± 0.66^d^	24.35 ± 0.55^b^
Ni + BEO	7.76 ± 0.10^b^	10.73 ± 0.28^e^	74.47 ± 0.37^e^	22.90 ± 0.33^b^
*P* value	< 0.001	< 0.001	< 0.001	< 0.001

Values not sharing the same superscript letter in the same column are significantly different (*P* < 0.05; one-way ANOVA; *n* = 8). *MDA*, malondialdehyde; *CAT*, catalase; *GSH*, reduced glutathione; *SOD*, superoxide dismutase. C (control), TTO, and BES: fish groups fed on basal, tea tree, and basil essential oils diets, respectively, without toxicant exposure. Ni, Ni + TTO, and Ni + BEO: fish groups exposed to 3.6 mg/L nickel and fed on basal, tea tree, and basil essential oils diets, respectively

### Immune indices and cortisol

The serum levels of MPO, LYZ, and NO were considerably elevated (*P* < 0.001) in the TTO and BEO groups comparable to the C group (Table 5). These immune variables were substantially declined in the Ni group in contrast to the C one. A considerable improvement in the MPO, LYZ, and NO was noticed in the Ni + TTO group and Ni + BEO group in contrast to the Ni group. Serum cortisol level was considerably lowered (*P* < 0.001) in the TTO and BEO groups, while elevated in the Ni group regarding the C group. This variable was substantially ameliorated in the Ni + TTO group accompanied by the Ni + BEO group regarding the Ni group.

**Table 5:** Immune indices and stress marker of Nile tilapia exposed to water-born nickel (Ni) and fed tea tree and basil essential oils for 45 days

	MPO (U/L)	LYZ (ng/mL)	NO (µmol/L)	Cortisol (mg/dL)
C	24.56 ± 0.42^b^	31.71 ± 0.59^b^	58.40 ± 2.40^b^	35.21 ± 0.51^d^
Ni	6.27 ± 0.20^d^	3.68 ± 0.37^d^	16.05 ± 1.57^c^	57.33 ± 0.54^a^
TTO	29.04 ± 0.47^a^	36.33 ± 0.27^a^	65.91 ± 2.11^a^	28.35 ± 0.62^e^
BEO	27.30 ± 0.39^a^	34.92 ± 0.03^a^	62.61 ± 3.59^a^	30.71 ± 0.42^e^
Ni + TTO	20.57 ± 0.45^c^	25.48 ± 0.39^c^	50.68 ± 2.28^b^	40.29 ± 0.61^c^
Ni + BEO	18.66 ± 0.54^c^	23.07 ± 0.11^c^	48.70 ± 3.70^b^	43.01 ± 0.41^b^
*P* value	< 0.001	< 0.001	< 0.001	< 0.001

Values not sharing the same superscript letter in the same column are significantly different (*P* < 0.05; one-way ANOVA; *n* = 8). *MPO*, myeloperoxidase; *LYZ*, lysozyme; *NO*, nitric oxide. C (control), TTO, and BES: fish groups fed on basal, tea tree, and basil essential oils diets, respectively, without toxicant exposure. Ni, Ni + TTO, and Ni + BEO: fish groups exposed to 3.6 mg/L nickel and fed on basal, tea tree, and basil essential oils diets, respectively

### Histopathological results

**Fig. 1:**
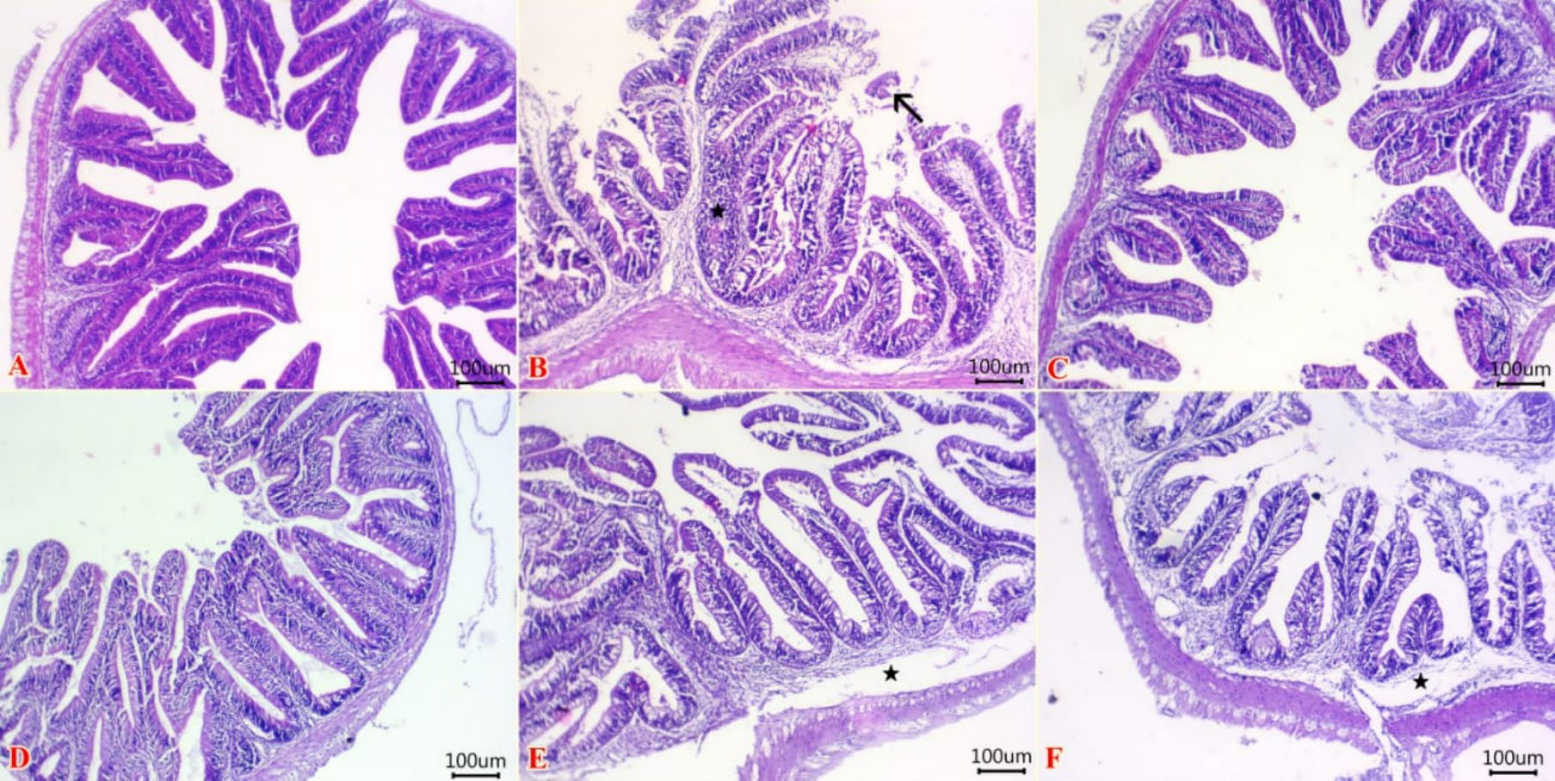
Photomicrograph of H&E stained sections from the intestine of fish showing **A** normal histological structures of mucosal epithelium, submucosa, and muscular layer in the C group, **B** inflammatory cell aggregations within lamina propria (star) beside detached and necrotic mucosal epithelium (arrow) in Ni group, **C** intact integrity of intestinal layers in TTO group, **D** normal intestinal layers with the presence of goblet cell hyperplasia in the BEO group, **E** edema with mild inflammatory cell infiltrations within lamina propria and submucosa (star) in Ni + TTO group, and **F** inflammatory edema within lamina propria and submucosa (star) beside metaplastic changes of columnar epithelial lining mucosa in the Ni + BEO group. Scale bar, 100 µm. C (control), TTO, and BES: fish groups fed on basal, tea tree, and basil essential oils diets, respectively, without toxicant exposure. Ni, Ni + TTO, and Ni + BEO: fish groups exposed to 3.6 mg/L nickel and fed on basal, tea tree, and basil essential oils diets, respectively

Normal histological structures of the intestine were represented by maintaining intact mucosal epithelium, submucosa, and the muscular layer was evident in the C group (Fig. 1A), while the intestinal tissue of the Ni group revealed necrotic enteritis represented by inflammatory cell aggregations within lamina propria beside detached and necrotic some mucosal epithelium (Fig. 1B). Interestingly, intact integrity of intestinal layers was demonstrated at TTO group (Fig. 1C). Normal intestinal layers with presence goblet cell hyperplasia were at BEO group (Fig. 1D). However, edema with mild inflammatory cells infiltrations within lamina propria and submucosa were seen at Ni + TTO group (Fig. 1E). Further, Ni + BEO group exhibited a mild degree of enteritis that represented by inflammatory edema within lamina propria and submucosa beside metaplastic changes of columnar epithelial lining mucosa (Fig. 1F).

**Fig. 2:**
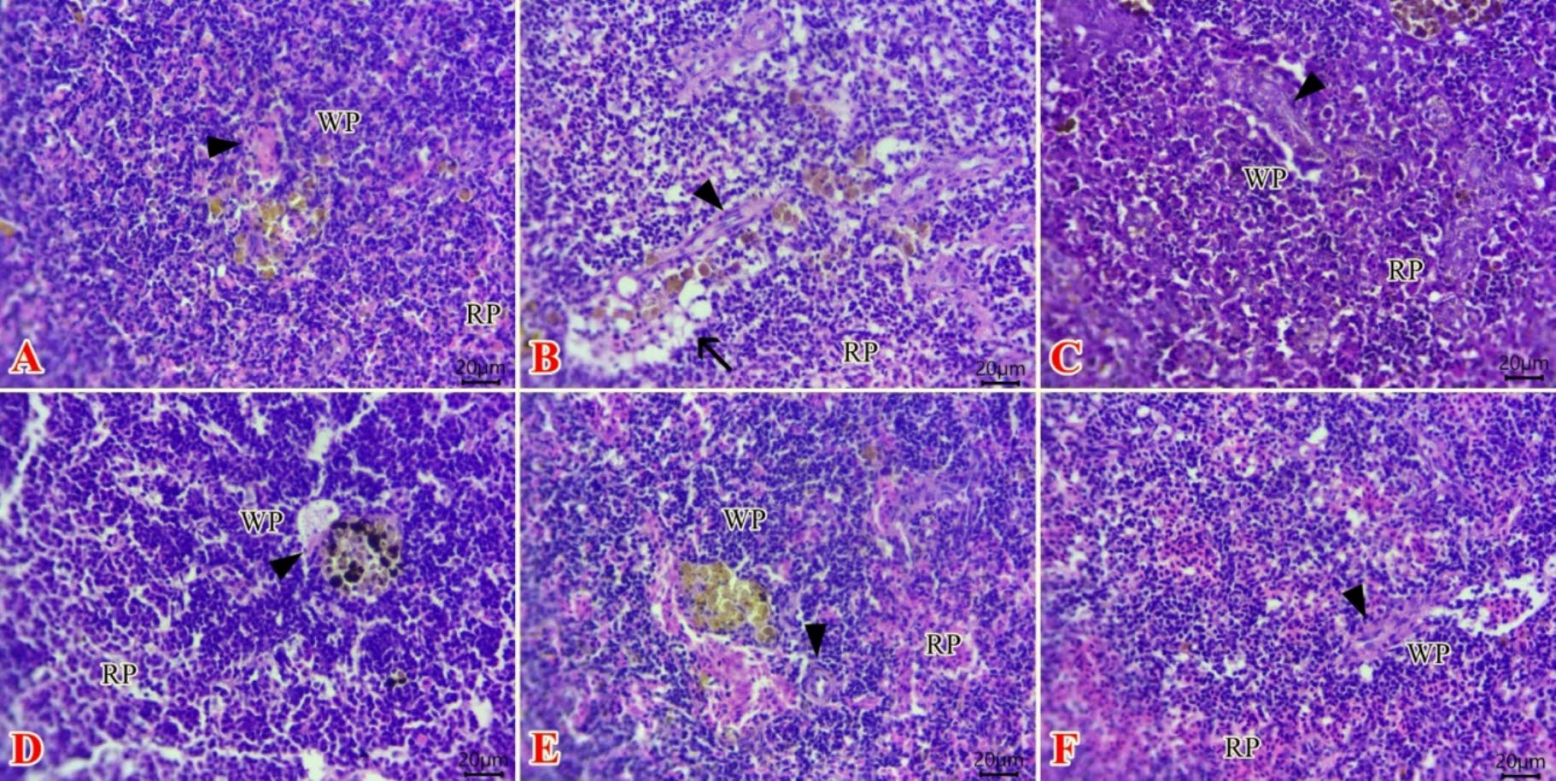
Photomicrographs of H&E stained sections of the spleen (**A**–**F**) from tilapia showing **A** normal histological structures of white pulp with melanomacrophages deposits around ellipsoids arterioles beside normal red pulp in the C group and **B** moderate depletion of lymphoid elements of white pulp (arrow) around ellipsoids arterioles in the Ni group, **C**, **D** apparently normal white pulp, central arterioles, melanomacrophage centers, and red pulp at both TTO and BEO groups, **E** re-established lymphoid elements in most white pulp with obvious melanomacrophage and dilated sinusoids in Ni + TTO group, **F** mildly vacuolation at some white pulps with preserved structures of most white pulps around ellipsoids arterioles that alternated with red pulps in the Ni + BEO group. RP (red pulp), WP (white pulp), central arterioles (arrowhead). Scale bar, 20 µm. C (control), TTO, and BES: fish groups fed on basal, tea tree, and basil essential oils diets, respectively, without toxicant exposure. Ni, Ni + TTO, and Ni + BEO: fish groups exposed to 3.6 mg/L nickel and fed on basal, tea tree, and basil essential oils diets, respectively

Normal histological structures of white pulp with melanomacrophages deposits around ellipsoids arterioles beside normal red pulp in the C group (Fig. 2A), while the Ni group (Fig. 2B) revealed moderate depletion of lymphoid elements of white pulp that appeared as empty spaces “either necrotic or apoptotic lymphocytes” around ellipsoids arterioles. However, apparently normal white pulp, central arterioles, melanomacrophage centers, and red pulp were observed in both TTO (Fig. 2C) and BEO group (Fig. 2D). On the other hand, re-established lymphoid elements in most white pulp with obvious melanomacrophage and dilated sinusoids were seen at Ni + TTO (Fig. 2E). Moreover, the Ni + BEO group (Fig. 2F) exhibited mildly vacuolation at some white pulps with preserved structures of most white pulps around ellipsoids arterioles that alternated with red pulps.

### Splenic gene expression

**Fig. 3:**
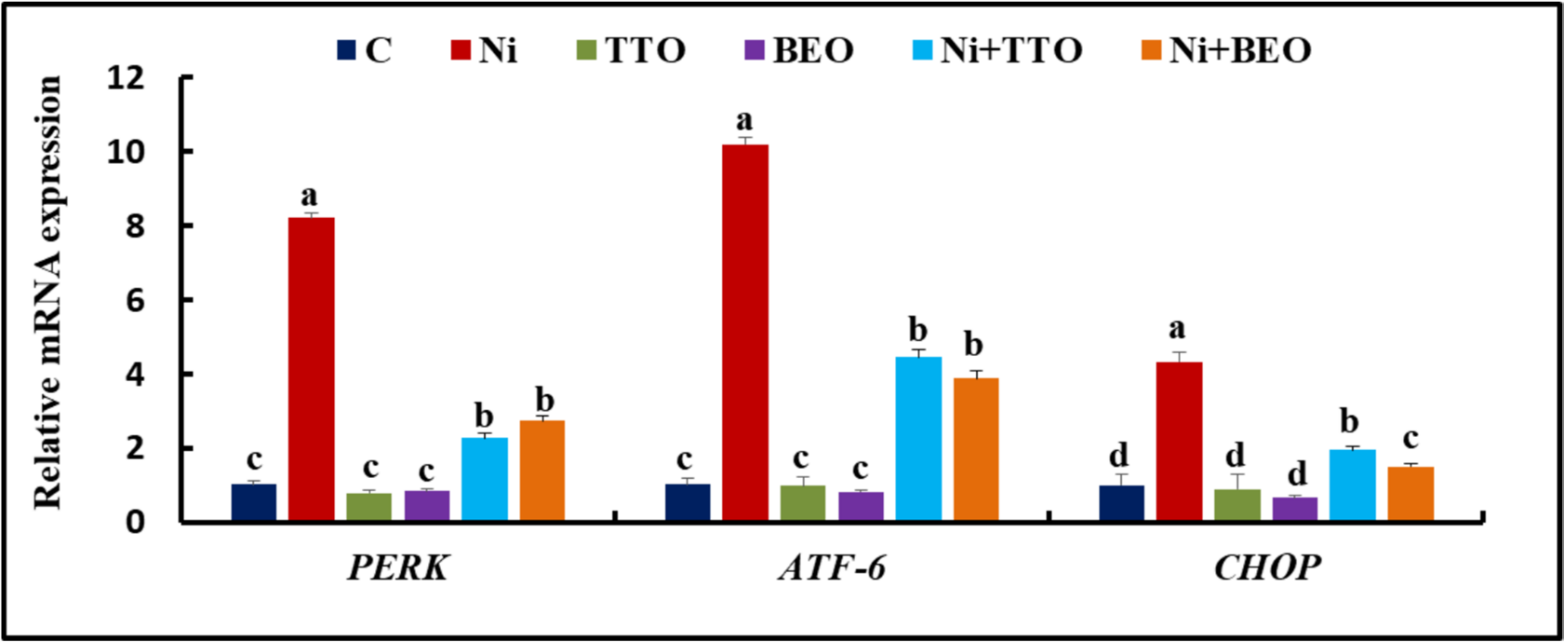
mRNA splenic expression of protein kinase R-like endoplasmic reticulum kinase (*PERK*), activating transcription factor 6 (*ATF-6*), and CCAAT/enhancer-binding protein homologous protein (*CHOP*) in Nile tilapia exposed to nickel intoxication and fed tea tree and basil essential oils-fortified diets. Values not sharing the same superscript letter in the same bar are significantly different (*P* < 0.05; one-way ANOVA; *n* = 8). C (control), TTO, and BES: fish groups fed on basal, tea tree, and basil essential oils diets, respectively, without toxicant exposure. Ni, Ni + TTO, and Ni + BEO: fish groups exposed to 3.6 mg/L nickel and fed on basal, tea tree, and basil essential oils diets, respectively

The splenic expression of *PERK*, *ATF-6*, and *CHOP* did not differ between the C, TTO, and BEO groups (Fig. 3), while their expression was substantially up-turned (*P* < 0.001) in the Ni group regarding the C group. In contrast to the Ni group, the *PERK*, *ATF-6*, and *CHOP* expression was considerably ameliorated in the Ni + TTO group accompanied by the Ni + BEO group regarding the *CHOP* expression, with no significant change between the later groups in the *PERK and ATF-6* expression.

**Fig. 4:**
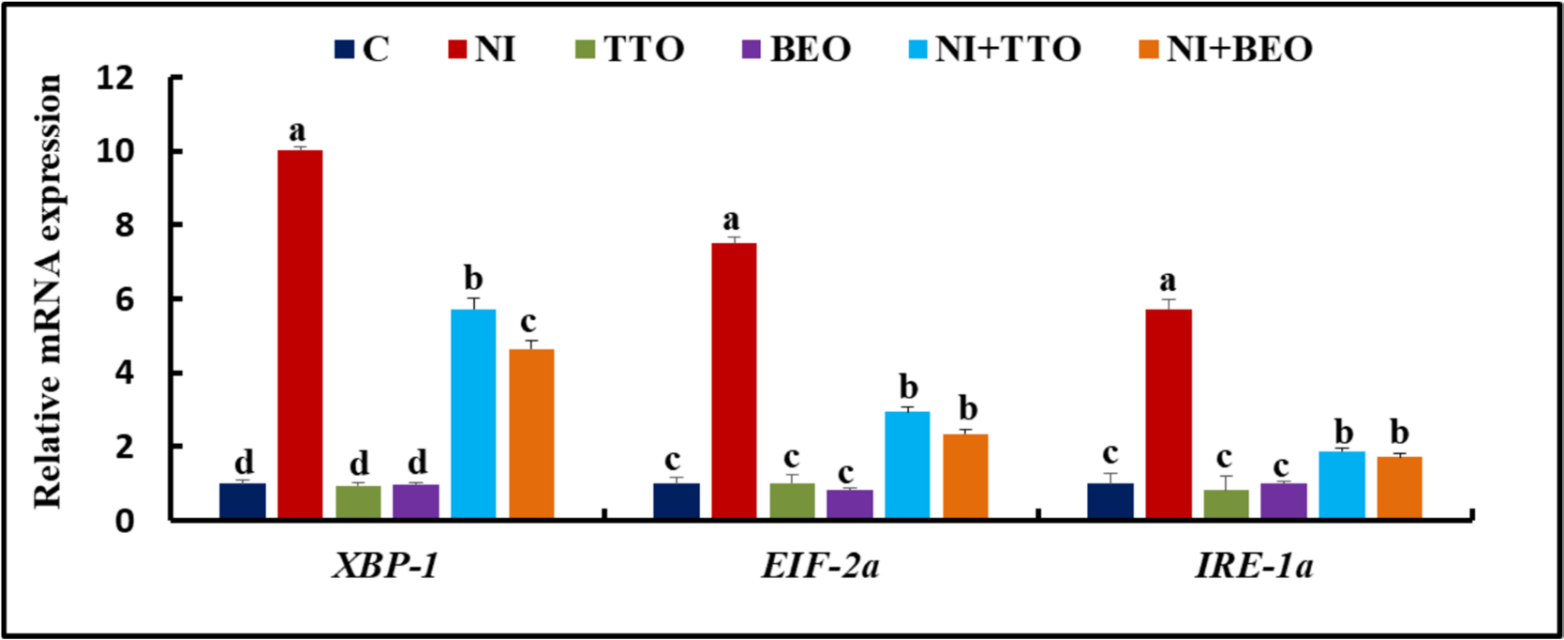
mRNA splenic expression of X-box binding protein 1(*XBP-1*), α-subunit of eukaryotic initiation factor 2 (*EIF-2a*), and *inositol*-requiring kinase 1a *(IRE-1a)* in Nile tilapia exposed to nickel intoxication and fed tea tree and basil essential oils-fortified diets. Values not sharing the same superscript letter in the same bar are significantly different (*P* < 0.05; one-way ANOVA; *n* = 8). C (control), TTO, and BES: fish groups fed on basal, tea tree, and basil essential oils diets, respectively, without toxicant exposure. Ni, Ni + TTO, and Ni + BEO: fish groups exposed to 3.6 mg/L nickel and fed on basal, tea tree, and basil essential oils diets, respectively

The expression of *XBP-1*, *EIF-2a*, and *IRE-1a* did not change between the C, TTO, and BEO groups (Fig. 4). Their expression was significantly elevated (*P* < 0.001) in the Ni group comparable to the C group. An amelioration of the expression of these genes was observed in the Ni + TTO group accompanied by the Ni + BEO group regarding *XBP-1* expression, with no significant change between the later groups in the *EIF-2a* and *IRE-1a* expression in contrast to the Ni group.

**Fig. 5:**
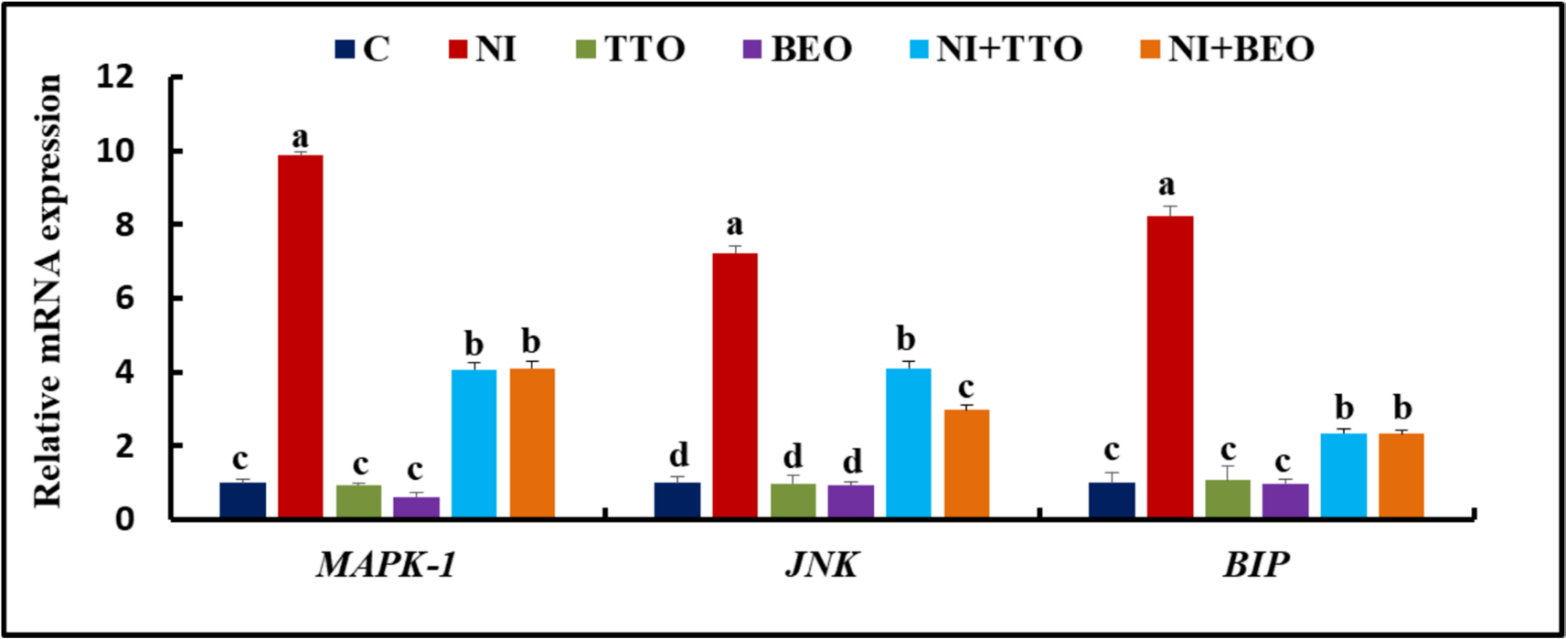
mRNA splenic expression of mitogen-activated protein kinase (*MAPK-1*), c-JunN-terminal kinase (*JNK*), and binding protein for immunoglobulins (*BIP*) in Nile tilapia exposed to nickel intoxication and fed tea tree and basil essential oils-fortified diets. Values not sharing the same superscript letter in the same bar are significantly different (*P* < 0.05; one-way ANOVA; *n* = 8). C (control), TTO, and BES: fish groups fed on basal, tea tree, and basil essential oils diets, respectively, without toxicant exposure. Ni, Ni + TTO, and Ni + BEO: fish groups exposed to 3.6 mg/L nickel and fed on basal, tea tree, and basil essential oils diets, respectively

The *MAPK-1*, *JNK*, and *BIP* expression levels did not significantly alter in the C, TTO, and BEO groups (Fig. 5). Up-regulation (*P* < 0.001) in the expression of these genes was detected in the Ni group comparable to the C group. A significant modulation in the expression of *MAPK-1*, *JNK*, and *BIP* was detected in the Ni + TTO accompanied by Ni + BEO groups regarding *JNK* expression, with no significant change between the later groups in the *MAPK-1* and *BIP* expression comparable to the Ni group.

## Discussion

Preventing the escape of heavy metals into the aquatic bodies is a challenge and difficult to be applied. Finding alternative strategies to combat the adverse consequences of such contaminants on fish health and immune systems can be applied. In this work, we explored the potential alleviative impacts of dietary TTO and BEO against water-born Ni exposure in Nile tilapia. Our results revealed that Ni exposure retarded the Nile tilapia growth. The reduced growth rate in the Ni-exposed fish could be related to reduced FI and increased FCR value as shown in this study. Additionally, the impaired growth could be related to the histopathological damage in the intestinal tissue of the Ni-exposed fish. Similar growth retardation was observed in Nile tilapia (Sheethal et al. 2024; El-Houseiny et al. 2022) and Zebrafish (*Danio rerio*) (Alsop et al. 2014) due to Ni exposure. Herein, in our trial, the growth of the Ni-exposed fish improved when fed on TTO and BEO diets. This could be attributed to enhanced tissue architecture of the intestinal tissue by feeding on the TTO and BEO diets. TTO improved the growth of *M. rosenbergii* (Liu et al. 2023) and Largemouth bass (*Micropterus salmoides*). The growth-promoting effect of TTO could be attributed to improved absorption capacity of nutrients and health of the intestinal tissue (Liu et al. 2022c). Similarly, BEO enhanced the growth performance of Nile tilapia (de Souza et al. 2019). The growth-promoting effect of BEO could be attributed to its components of Linalool and eucalyptol (Souza et al. 2018b).

Fish may exhibit oxidative stress brought on by ROS, which could disrupt physiological and biochemical processes (Karayakar et al. 2022). To identify the sub-lethal implications of metals on fish metabolisms, a number of indicators were used. Thus, these biomarkers play a “prompt caution mechanism” role for metal exposure before toxic consequences or death occurs (Karayakar et al. 2022; Eroglu et al. 2015). Consequently, in fish, antioxidant system indicators are frequently employed for this objective (Atli and Canli 2010). It is commonly known that metal ions raise ROS, which changes the amounts of various molecules or the activities of numerous enzymes. Additionally, metals can tip the antioxidant defense systems’ negative balance toward oxidants, which can lead to oxidative stress (Kanak et al. 2014). Cells contain both enzymatic and non-enzymatic antioxidant components to fight oxidative stress caused by a variety of stressors. The two most important enzymes in the antioxidant system are CAT and SOD, while non-enzymatic antioxidants include compounds like GSH. Research revealed that organisms exposed to xenobiotics experienced increases in MDA levels, indicating that these exposures had detrimental effects on cell structure (Karayakar et al. 2022; Canli and Canli 2024). In this trial, the Ni exposure induced oxidative stress in the Ni-exposed fish through inhibition of the CAT, SOD, and GSH activity and elevated MDA level. Similar outcomes were previously documented in Nile tilapia as a result of Ni exposure (El-Houseiny et al. 2022). Such oxidative stress could be attributed to the produced ROS by Ni which suppresses the antioxidant activity (Salnikow et al. 1994). In this trial, feeding on the TTO and BEO diets improved the antioxidant capacity and reduced the oxidative stress in the Ni-exposed fish. TTO has active components such as terpinene-4-ol, γ-terpinene, and α-terpinene which have antioxidant activity (Souza et al. 2018a). Dietary TTO at a level of 0.1% reduced the ROS level and lipid damage caused by amitraz exposure in *R. quelen* (dos Reis et al. 2021). BEO may have antioxidant attributes because it contains vitamins, carotenoids, flavonoids, and essential oils (e.g., eugenol, methyl chavicol, methyl cinnamate, and linalool), all of which have been shown to have major health benefits by reducing oxidative harm (Ahmed et al. 2019).

According to the results of the current work, fish exposed to nickel experienced immunosuppression as a result of decreased MPO, LYZ, and NO levels. One of the essential components of the immune response is MPO, a glycosylated, heme-containing, highly cationic enzyme (Odobasic et al. 2016). MPO helps the phagosomes maintain an alkaline environment, which is ideal for the activity of other granule components and serine proteases to inactivate and kill microorganisms (Arnhold 2020). MPO catalyzes the production of ROS in the presence of halides and hydrogen peroxide, which helps neutrophils kill microorganisms (Aratani 2018). LYZ are innate immune components that have crucial impacts on fish immune responses against pathogens (Li et al. 2021b; Shen et al. 2021). The LYZ level responds to infection, stress, and nutritional factors (Castanho et al. 2017; Magnadottir 2010). NO is an essential molecule of the fish’s innate immune responses and has antimicrobial activity (Rodríguez-Ramos et al. 2016).

Herein, suppression of the immune indices in the Ni-exposed fish could be related to elevated cortisol levels which was evident in our work. According to Campbell et al. (2021), chronic stress elevates cortisol secretion which leads to the inhibition of the immune cytokines, inhibits the leucocyte differentiation and distribution, and finally lowers the innate immune responses. Furthermore, the study’s detection of immune response inhibition may be connected to the histological alterations in spleen tissue brought on by Ni exposure Oxidative stress results in over-ROS production which induces tissue architecture damage and finally alters its function (Altun et al. 2017).

In this study, an improvement of the immune responses and spleen architecture was observed in the Ni-exposed fish by feeding on the TTO and BEO diets. TTO has limonene and 1,8-cineole components which exert immunostimulant properties through the improvement of the leucocytes and LYZ in rainbow trout (Gültepe 2020). Additionally, TTO has α-terpineol and γ-terpinene which exerts immunostimulant properties through improvement of the blood leucocytes in African carp (*Labeo victorianus*) (Ngugi et al. 2017). Flavonoids, total phenolics, cineole, terpineol, and linalool are among the many phytochemically active constituents found in BEO, and these substances are thought to support immunological function (Ahmed et al. 2020). El-Ashram et al. (2017) reported that *O. niloticus* fed a diet fortified with BEO showed enhanced immune function indicators and enhanced immunological response. One of the most significant organelles in fish, the endoplasmic reticulum (ER), is intimately linked to cell homeostasis. The ER is a molecular mechanism elaborated due to cellular stress such as exposure to toxic substances, starvation, and oxidative stress, this pathway aims to conserve the cellular energy to the vital function via inhibiting the mRNA translation process via the *EIF-2α/IRE-1α/ATF-6* pathway and/or splicing the mRNA via the activation of the spliceosome (*XBP-1*). The prolonged stimulation of these pathways leads to the overexpression of CHOP that subsequently activates the intrinsic and extrinsic apoptotic pathways (Khamis et al. 2021; Schröder and Kaufman 2005). On the other hand, ER stress is associated with a perturbation in the ER calcium/ATPase pump (SERCA), which is associated with the misfolded and unfolded protein response (UPR). The mis/unfolded protein accumulated in the cytoplasm and served as an autoantigen that in turn flared a marked inflammatory condition (Osorio et al. 2014). Based on the previous notion, the dysfunction of the intestinal barriers and the marked immune suppression recorded in the findings of the current work might be attributed to the inhibition of the protein expression of tight junction elements (Yong et al. 2022), inflammatory conditions, and the marked induction of the apoptosis process in response to the overexpression of the CHOP considered a potent driver for the inflammatory and the apoptotic pathways (Cao et al. 2022). In this study, Ni-exposure activated the *PEPK*/*ATF-6*/ *IRE-1-a* signaling pathways with increased core gene expression of *PERK*, *ATF-6*, *CHOP*, *XBP-1*, *EIF-2a*, *IRE-1a*, *MAPK-1*, *JNK*, and *BIP* indicating that Ni exposure induced ER stress. In similar, Li et al. (2021a) showed that cadmium toxicity in Gibel carp (*Carassius gibelio*) can cause ER stress in fish by altering the expression of *IRE-1*, *PERK*-, and *ATF6*-signaling pathway. *PERK* is the primary translational control regulator during the UPR (Harding et al. 2000b). *ATF-6* can activate the expression of the *CHOP* which is considered a pro-apoptotic gene (Harding et al. 2000a). Additionally, *XBP-1* and *EIF-2a* mediated the activation of *CHOP* (Yao et al. 2023). Through the control of genes linked to the cell cycle and the production of ROS, *CHOP* has been implicated in numerous processes that result in cellular apoptosis (Tapia-Limonchi et al. 2014). *IRE-1a* triggers *JNK*, which induces *CHOP* expression to suppress the anti-apoptotic genes and activate apoptosis pathways (Tomicic et al. 2015; Almanza et al. 2019). During ER stress, *BIP* expression is up-regulated and considered a crucial indicator of ER stress (Walter and Ron 2011). Fish innate immunity and inflammatory responses are mediated by conserved signaling cascades called *MAPK* pathways, which are triggered by environmental stressors (Umasuthan et al. 2015). Both *MAPK* and *JNK* were triggered by excessive ROS (Duan et al. 2022). The Ni-exposed fish when fed on the TTO and BEO diets exhibited an amelioration of the ER stress-associated genes. Interestingly, the results showed that dietary supplementation with TTO and BEO induced a marked inhibition of the ER stress markers thus improving the synthesis of the tight junction proteins, abrogating the inflammatory cascade, and subsequently reducing the intestinal and splenic apoptotic process that is in line with Yong et al. (2022). Reducing the ER stress as a result of TTO and BEO could be attributed to their antioxidant activity and reducing oxidative stress and ROS production. Therefore, it is essential to lower oxidative stress and ER stress in order to enhance fish immunity and health (Liang et al. 2022). Overall, TTO and/or BEO could enhance the antioxidant status and immune capacity in Nile tilapia, consequently reducing the ER stress as a result of Ni-exposure. Noteworthy, the effect of the TTO diet was more pronounced than the BEO diet.

## Conclusion

Ni-exposure in Nile tilapia induced the growth performance of Nile tilapia through reduced FI and increased FCR. Additionally, Ni-exposure initiated oxidative stress and immune suppression by lowering the CAT, GSH, SOD, MPO, LYZ, and NO activity and increased MDA levels. ER stress was initiated by Ni-toxicity through activation of the *PEPK*/*IRE-1-a*/*ATF-6*-signalling pathways in the exposed fish. Dietary fortification of TTO and/or BEO improved the growth performance, antioxidant capacity, and immune responses of the Ni-exposed fish. Additionally, reduction of the ER stress signals was attained by feeding the Ni-exposed fish on TTO and/or BEO diets. The benefits of TTO dietary fortification were more evident than the BEO one.

## Supplementary Information

Below is the link to the electronic supplementary material.

ESM 1 (DOCX 131 KB)

## Data Availability

No datasets were generated or analysed during the current study.
