# Supplementary material for: The ability of dietary essential oils to mitigate nickel-induced growth retardation, immune-antioxidant suppression, and endoplasmic reticulum stress activation in Nile tilapia
Source: Fish Physiol Biochem. 2025 Mar 31;51(2):76. doi: 10.1007/s10695-025-01482-2 (PMC11958502; doi:10.1007/s10695-025-01482-2)
Supplement: Supplementary file 1 — (DOCX 131 KB) [file 10695_2025_1482_MOESM1_ESM.docx]

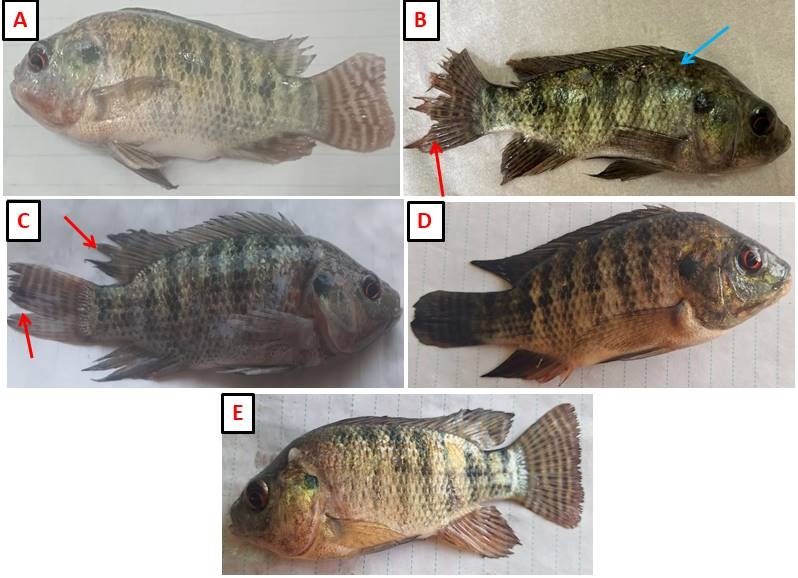


Clinical signs of Nile tilapia exposed to nickel-intoxication and fed on tea tree (TTO) and basil essential oils (BEO) -fortified for 45 days. A representative photo to control, TTO, and BEO groups exhibit a normal appearance. B−C Fish that were exposed to 3.6 mg/L nickel, exhibit skin darkness (blue arrows) and fin rot . D−E Fish exposed to nickel and fed on TTO and BEO diets, respectively exhibit apparently normal appearance.
